# *Pristimantis achupalla* sp. n., a new minute species of direct-developing frog (Amphibia, Anura, Strabomantidae) inhabiting bromeliads of the montane forest of the Amazonian Andes of Puno, Peru

**DOI:** 10.7717/peerj.11878

**Published:** 2021-09-23

**Authors:** Alex Ttito, Alessandro Catenazzi

**Affiliations:** 1 Departamento de Ecología, Pontificia Universidad Católica de Chile, Santiago, Chile; 2 Museo de Biodiversidad del Perú, Cusco, Perú; 3 Museo de Historia Natural del Cusco, Universidad Nacional San Antonio Abad del Cusco, Cusco, Perú; 4 Department of Biological Sciences, Florida International University, Miami, Florida, United States; 5 Centro de Ornitología y Biodiversidad, Lima, Perú; 6 Instituto Peruano de Herpetología, Lima, Perú

**Keywords:** Frog, Taxonomy, 16S rRNA, Andes, Carabaya, Cloud forest, Bromeliads

## Abstract

We describe a new species of bromeliad-dwelling *Pristimantis* from primary montane forest (2,225 m a.s.l.) in southern Peru. The type locality is near Thiuni, in the Department of Puno (province of Carabaya) in the upper watershed of a tributary of the Inambari River. *Pristimantis achupalla* sp. n. is characterized by a snout-vent length of 10.0–12.8 mm in adult males (*n* = 4), unknown in adult females, and is compared morphologically and genetically with species in the *Pristimantis lacrimosus* group, and with other similar species of *Pristimantis*. The new species is characterized by having skin on dorsum and flanks rugose, green brownish color, distinctive scapular folds, subacuminate or acuminate snout profile, upper eyelid bearing two or three subconical tubercles and some rounded tubercles, rostral papilla, flanks light brown to brown, with irregular dark brown marks.

## Introduction

The neotropical amphibian genus *Pristimantis* (Anura, Terraranae, Strabomantidae) is the most speciose genus of terrestrial vertebrates; *Pristimantis* frogs are often important components of ecological communities in terms of both species composition and individual abundance (Padial, Grant & Frost, 2014). Among approximately 569 currently described species (Frost, 2021), 142 species of *Pristimantis* are currently known from Peru (Chávez, García-Ayachi & Catenazzi, 2021). New species are described every year, mainly from the Andes (Lehr et al., 2017; Catenazzi & Lehr, 2018).

The remarkable diversity of this terrestrial group may be associated with the success of their reproductive mode. By reproducing through direct development, individuals can be independent of water, and they can colonize new terrestrial niches (Hedges, Duellman & Heinicke, 2008). It is thought that this ability to colonize and establish populations away from water promoted the rapid accumulation of population genetic isolation across the landscape (Elmer, Davila & Lougheed, 2007; Fouquet et al., 2007). Our knowledge of the diversity of this genus is thus still incomplete, and the rate of species discovery remains high throughout the Andes, including in eastern Peru, where several species have recently been described (*e.g.*, Shepack et al., 2016; Chávez & Catenazzi, 2016; Padial et al., 2016; Catenazzi & Lehr, 2018).

**Figure 1:**
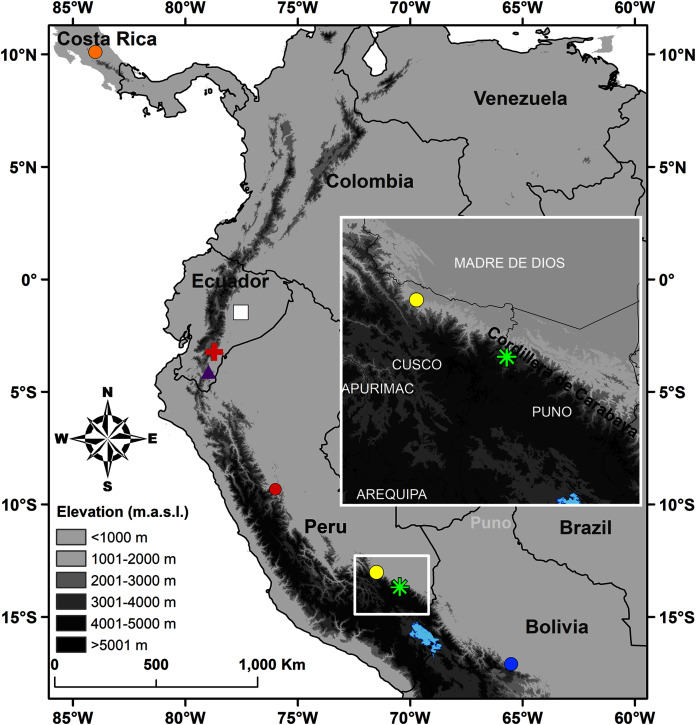
Map of Peru indicating the type locality of *Pristimantis achupalla* sp. n. (green asterisk). The most closely related species according to our phylogeny, *Pristimantis* sp. CORBIDI 12183 (yellow point), *P. amaguanae* (white square), *P. moro* (orange point), *P. bromeliaceus* (red cross), *Pristimantis* sp. QCAZ 60398 (purple triangle) from Ecuador, and of other Peruvian species closest geographically in the *Pristimantis lacrimosus* group: *P. pluvialis* (yellow point) Kosñipata, Cusco, *P. pulchridormientes* (red point) from Tingo Maria, Huánuco, and *P. olivaceus* (blue point) from Cochabamba, Bolivia.

Here we describe a new species of *Pristimantis* found in the bromeliads of a cloud forest remnant in the Cordillera de Carabaya (Fig. 1), in the southern Peruvian department of Puno, along a tributary of the Inambari River. This specimen does not resemble any of the previously described species of *Pristimantis*. Furthermore, molecular data indicate that the sequence of a fragment of 16S rRNA does not match any known sequence of *Pristimantis*. The species is most closely related to an undescribed species from cloud forests in Cusco, Peru. On the basis of these lines of evidence, here we describe the species.

## Materials & methods

During a rapid survey at a primary cloud forest (2,220 m a.s.l.) near Thiuni village, Ollachea Valley, department of Puno in southern Peru, on 14 August 2017, we collected specimens of *Pristimantis* frogs by opportunistic inspections in epiphytes bromeliads. The individuals sampled were photographed alive and euthanized with 20% benzocaine. Samples of tissues were extracted from some specimens and preserved in 96% ethanol for molecular analysis. Specimens were fixed in 10% formalin and stored in 70% ethanol. We deposited specimens in the herpetological collection at Centro de Ornitologia y Biodiversidad (CORBIDI) and Herpetology Department, Museo de Biodiversidad del Peru (MUBI). We conducted this research under collection permits (permits #0292-2014-MINAGRI-DGFFS/DGEFFS, #029-2016-SERFOR-DGSPFS) issued by The Dirección General Forestal y de Fauna Silvestre, Ministerio de Agricultura y Riego. The protocol of collection and animal care of our research was approved by the Institutional Animal Care and Use Committee of Florida International University (protocol #18-009).

We wrote the definition (morphological characterization of the new species) following the standard format of Duellman & Lehr (2009). The description follows Lynch & Duellman (1997). We follow Heinicke et al. (2018) for family placement and taxonomy classification. Under a stereomicroscope and using a caliper, we measured the following variables to the nearest 0.1 mm, as described by Duellman & Lehr (2009): snout–vent length (SVL), head length (HL, obliquely from angle of jaw to tip of snout), head width (HW, at level of angle of jaw), eye diameter (ED), tympanum diameter (TD), interorbital distance (IOD), upper eyelid width (EW), internarial distance (IND), and eye–nostril distance (E–N, straight line distance between anterior corner of orbit and posterior margin of external nares), forearm length (FAL), hand length (HAL, distance from proximal edge of palmar tubercle to the tip of Finger III), tibia length (TL), foot length (FL, distance from proximal margin of inner metatarsal tubercle to tip of Toe IV). Fingers and toes are numbered preaxially to postaxially from I–IV and I–V respectively. We compared the lengths of toes III and V by adpressing both toes against Toe IV; lengths of fingers I and II were determined by adpressing the fingers against each other. We used field notes and photographs we took in the field to describe coloration in life. In addition to the type series of the new species, we obtained morphological data from the original descriptions and examined specimens of related congeners (Appendix I) for comparisons of diagnostic characters. See Table 1 for morphometric measurements of the type series.

**Table 1:** Measurements (in mm) of holotype and paratopotypes of *Pristimantis achupalla* sp. n. from Thiuni, 2225 m a.s.l., Distrito Ollachea, Provincia Carabaya, Department of Puno, Peru.

Characters	Holotype male	Paratypes (all males)		
	CORBIDI 18736	CORBIDI 18737	MUBI 17604	MUBI 17605
SVL	12.8	11.7	10.0	10.4
Head length	5.1	4.8	4.5	4.6
Head width	4.6	4.2	4.0	3.9
Eye diameter	1.3	1.3	1.2	1.1
Tympanum diameter	0.3	0.2	0.25	0.25
Interorbital distance	1.8	1.6	1.6	1.6
Upeer eyelid width	1.3	1.0	1.0	0.9
Internarial distance	1.2	1.0	1.0	1.0
Eye-nostril distance	1.1	1.0	0.9	0.9
Tympanum diameter	0.3	0.2	0.25	0.25
Eye to tympanum distance	0.3	0.2	0.2	0.2
Foream length	2.5	2.3	2.1	2.1
Hand length	3.4	2.9	2.6	2.8
Tibia length	6.5	4.3	5.2	5.3
Foot length	5.2	4.8	4.2	4.2
HL/SVL	0.4	0.4	0.5	0.4
HW/SVL	0.4	0.4	0.4	0.4
FL/SVL	0.4	0.4	0.4	0.4
FL/TL	0.8	1.1	0.8	0.8
HW/HL	0.9	0.9	0.9	0.8
EW/IOD	0.7	0.6	0.6	0.6

We used phylogenetic analyses to examine relationships between the new species and other species of *Pristimantis*. We sequenced a fragment of the 16S rRNA mitochondrial gene. 16S rRNA is the gene most commonly amplified gene for anuran and Terrarana taxonomy (Fouquet et al., 2007; Hedges, Duellman & Heinicke, 2008; Padial & De la Riva, 2009; Vences et al., 2005), and the gene with the largest number of available sequences on Genbank. We used liver tissues from two paratypes of the new species to obtain DNA sequences (accession codes MW724812 and MW724813; Appendix II). Additionally, we obtained DNA sequences from samples of described and undescribed *Pristimantis* species collected in southern Peru (Cusco and Madre de Dios) (Shepack et al., 2016). Finally, we downloaded sequences from Genbank (Appendix II) of morphologically similar species in the putative *P. lacrimosus* group (*sensu* Arteaga, Yáñez-Muñoz & Guayasamin, 2013; Rivera-Correa & Daza, 2016; 2020; Ron et al., 2020), along with representative samples of other species groups of *Pristimantis*. We followed standard protocols of DNA extraction, amplification, and DNA sequencing (Hedges, Duellman & Heinicke, 2008; Catenazzi & Ttito, 2016). For amplification, we used the primers16Sar (forward: 5′-CGCCTGTTTATCAAAAACAT-3′) and 16Sbr (reverse: 5′-CCGGTCTGAACTCAGATCACGT-3′) (Palumbi et al., 2002) and the following polymerase chain reaction thermocycling conditions (Proflex thermal cycler, Applied Biosystems): one cycle at 96 °C/3 min; 35 cycles at 95 °C/30 s, 55 °C/45 s, 72 °C/1.5 min; and one cycle at 72 °C/7 min. We used Exosap-IT (ThermoFisher) to purify PCR products. MCLAB (South San Francisco, CA, USA) performed sequencing. We used AliView version 1.26 (https://ormbunkar.se/aliview/) to align sequences with the MUSCLE v3.8.31 alignment program (Edgar, 2004) and trimmed sequences to a length of 1623 bp. Our analysis included 86 terminals representing 61 nominal species within *Pristimantis* and one terminal as outgroup *Niceforonia dolops*. We inferred the best evolution model using ModelFinder under the BIC criterion (Kalyaanamoorthy et al., 2017). We employed a Maximum Likelihood (ML) as optimality criterion to infer a molecular phylogeny generated using W-IQ-TREE web server (Trifinopoulos et al., 2016) based on the best model inferred TIM2 + F + I + G4. To assess node support, we made 1,000 replicates for the SH-like approximate likelihood ratio test (SH-aLRT, Guindon et al., 2010) and 1,000 ultrafast bootstrap (UFBoot2, Hoang et al., 2018). We considered that nodes with SH-aLRT values ≥80 and ultrafast bootstrap values ≥95 had strong support, also (SH-aLRT ≤ 50% and UFBoot ≤ 70% slightly overestimate the true probability, Minh, Nguyen & Von Haeseler, 2013). We also estimated pairwise, uncorrected genetic distances (*p*-distances) for 16S rRNA between the new species and the closest related species (*i.e., Pristimantis amaguanae, P. bromeliaceus, P. moro, Pristimantis* sp. QCAZ 60398, *Pristimantis* sp. CORBIDI 12183) and the closest geographical species of *P. lacrimosus* species group (*i.e.*, *P*. cf. *olivaceus, P. pluvialis, and P. pulchridormientes*) using the software MEGA 7 (Kumar, Stecher & Tamura, 2016).

### Nomenclatural act

The electronic version of this article in Portable Document Format (PDF) will represent a published work according to the International Commission on Zoological Nomenclature (ICZ), and hence the new name contained in the electronic version is effectively published under that Code from the electronic edition alone. This published work and the nomenclatural acts it contains have been registered in ZooBank, the online registration system for the ICZN. The ZooBank LSIDs (Life Science Identifiers) can be resolved and the associated information viewed through any standard web browser by appending the LSID to the prefix http://zoobank.org/. The LSID for this publication is: urn:lsid:zoobank.org:pub:88FB4422-449E-45BF-B2E3-0A30D00090B8. The online version of this work is archived and available from the following digital repositories: PeerJ, PubMed Central and CLOCKSS.

## Results

### Generic placement

**Figure 2:**
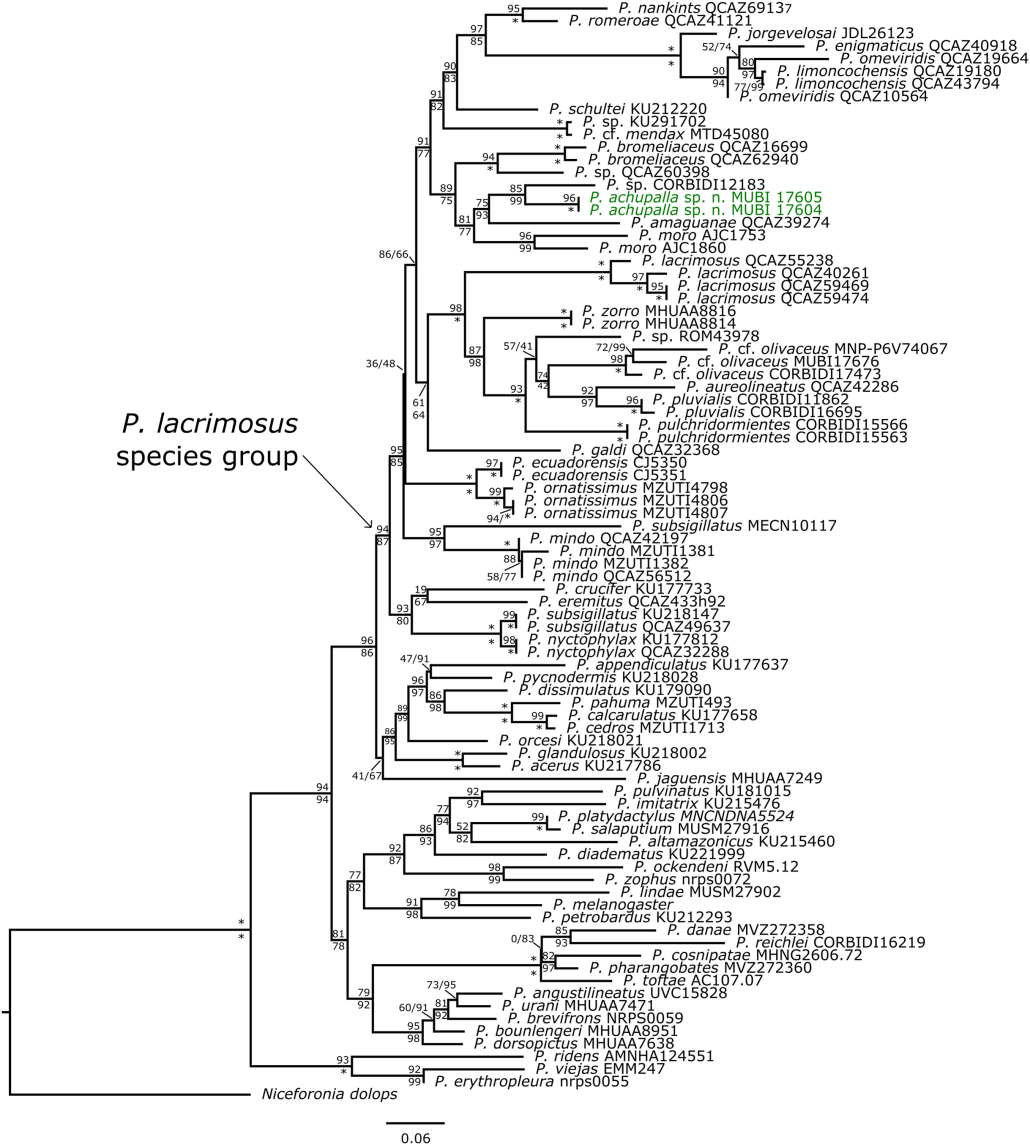
Phylogenetic analysis of 16S rRNA by using Maximum Likelihood. SH-aLRT support (above) and ultrafast bootstrap support (below) are shown as percentages on branches. Asterisks indicate support values of 100 (bootstrap). Species and Voucher number of the samples are shown next to each terminal. The new species is shown with bold green characters. The outgroup is *Niceforonia dolops*.

We tentatively allocate *P. achupalla* sp. n. to the putative *P. lacrimosus* group *sensu* Arteaga, Yáñez-Muñoz & Guayasamin (2013) due to the presence of rostral tubercle, acuminate snout profile, distinct tympanic membrane, moderately long limbs, Finger I shorter than Finger II, and expanded digital disks. Furthermore, according to our phylogeny (Fig. 2), the new species is nested in a clade that includes the species of the *Pristimantis lacrimosus* group (clade B, *sensu* Rivera-Correa & Daza, 2016). The topology for the sampled *Pristimantis* includes nodes whit moderate to strong support and is similar to topologies of previous phylogenies (Arteaga, Yáñez-Muñoz & Guayasamin, 2013; Padial, Grant & Frost, 2014; Rivera-Correa & Daza, 2016; Rivera-Correa & Daza, 2020; Ron et al., 2020). The new species is the sister taxon of a putative new species with high support, and both are related to recently described species *P. amaguanae* from Ecuador (see Ron et al., 2020), and the known species *P. moro* from Panama. Furthermore, these taxa form a small clade that is sister to a small clade composed of *P. bromeliaceus* and one undescribed species *P*. sp. QCAZ62940 (Fig. 2). We found substantial genetic distances (uncorrected *p*-distances of 0.05–0.11, Table 2) between *P. achupalla* and the most closely related species. *Pristimantis achupalla* is most closely related to one undescribed *Pristimantis*, from Yanamayo, Kosñipata Valley in Cordillera Vilcanota approximately 145 km NE of the type locality (paratypes MUBI 17604 05, 16S uncorrected *p*-distance: 0.05). Other closely related species are *P. amaguanae* (0.06–0.07) Pastaza, Ecuador; *P. moro* (0.07–0.09) from Chilibre and Darien, Panama; *P. sp*. QCAZ 60398 (0.07) Bombuscaro, Zamora Chinchipe, Ecuador; *P. bromeliaceus* (0.10–0.11) from Morona and Zamora respectively, Ecuador.

**Table 2:** Uncorrected *p*-distances for the fragment of the mitochondrial 16S rRNA gene.

Taxon		1	2	3	4	5	6	7	8	9	10	11	12	13	14	15	16
1	*P. bromeliaceus* QCAZ 16699 ECU																
2	*P. bromeliaceus* QCAZ 62940 ECU	0.04															
3	*P*. sp. QCAZ 60398 ECU	0.10	0.12														
4	*P. moro* AJC1753 PAN	0.09	0.10	0.09													
5	*P. moro* AJC1860 PAN	0.08	0.09	0.07	0.05												
6	*P. amaguanae* QCAZ39274 ECU	0.20	0.20	0.20	0.07	0.07											
7	*P*. sp. CORBIDI12183 PER	0.10	0.10	0.07	0.10	0.08	0.08										
8	*P. achupalla* sp. n. MUBI 17604 PER	0.10	0.11	0.07	0.09	0.07	0.06	0.05									
9	*P. achupalla* sp. n. MUBI 17605 PER	0.11	0.12	0.07	0.09	0.07	0.07	0.05	0.00								
10	*P. pulchridormientes* CORBIDI15566 PER	0.16	0.16	0.09	0.11	0.11	0.08	0.12	0.12	0.14							
11	*P. pulchridormientes* CORBIDI15563 PER	0.16	0.16	0.09	0.11	0.11	0.08	0.12	0.12	0.14	0.00						
12	*P. pluvialis* CORBIDI11862 PER	0.11	0.11	0.10	0.12	0.09	0.08	0.10	0.11	0.11	0.07	0.07					
13	*P. pluvialis* CORBIDI16695 PER	0.17	0.17	0.10	0.12	0.09	0.08	0.10	0.12	0.17	0.11	0.11	0.00				
14	*P. cf. olivaceus* NMP-P6V74067 BOL	0.18	0.17	0.10	0.12	0.10	0.10	0.12	0.12	0.17	0.12	0.12	0.08	0.10			
15	*P*. cf. *olivaceus* CORBIDI17473 PER	0.15	0.15	0.10	0.11	0.09	0.09	0.11	0.11	0.15	0.10	0.10	0.06	0.10	0.05		
16	*P*. cf. *acuminatus* CORBIDI17676 PER	0.11	0.10	0.09	0.10	0.09	0.08	0.12	0.11	0.11	0.08	0.08	0.07	0.07	0.04	0.03	

**Note:**

Comparisons between *P. achupalla* and the closest related species, and other closest geographical species within *P. lacrimosus* group.

#### *Pristimantis achupalla* sp. n.

urn:lsid:zoobank.org:act:D9EFDD3D-F0C3-40D8-BEBF-71372B43D42D; urn:lsid:zoobank.org:pub:88FB4422-449E-45BF-B2E3-0A30D00090B8.

### Holotype

**Figure 3:**
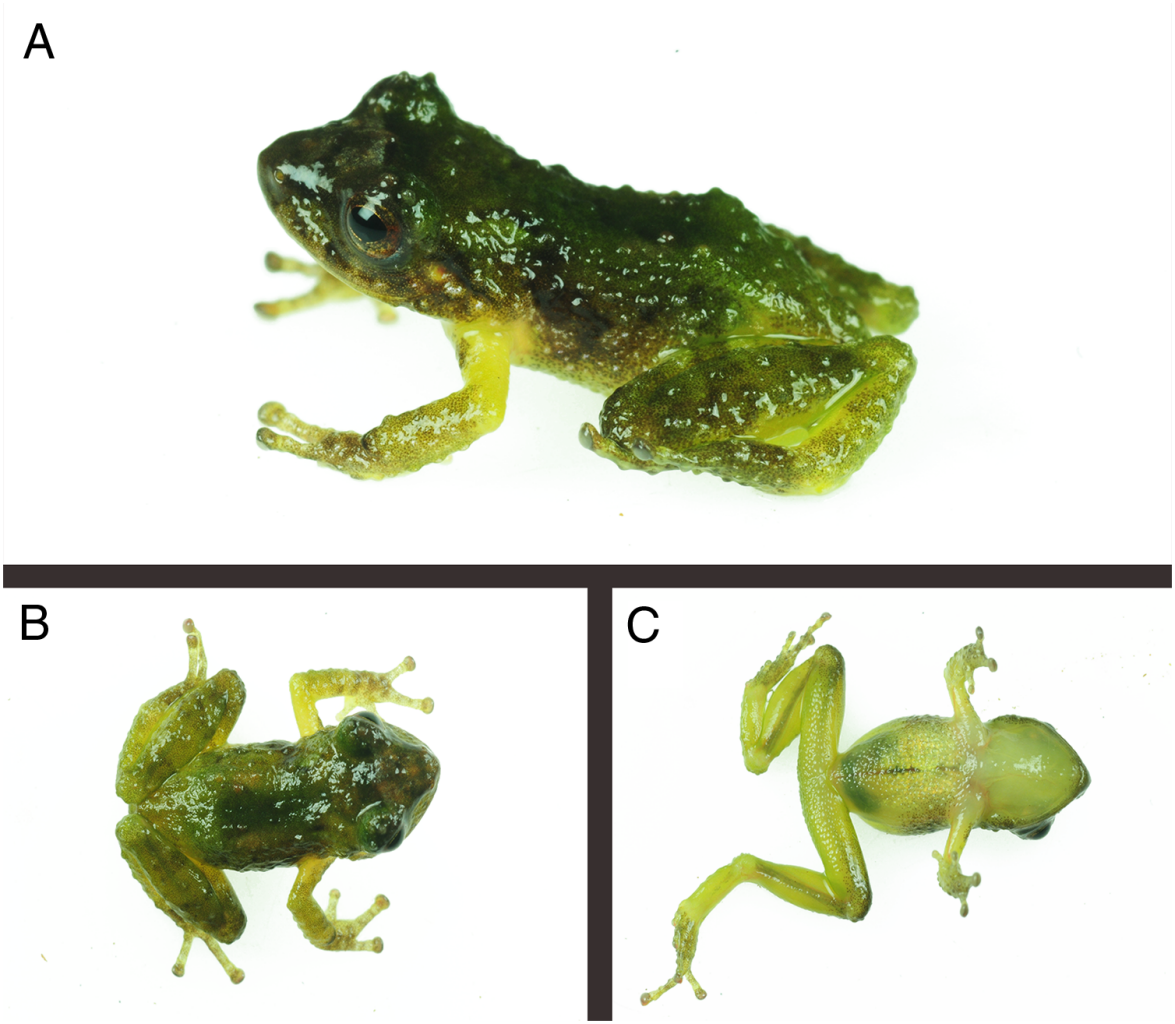
Holotype of *Pristimantis achupalla* sp. n., male CORBIDI 18736 (SVL 12.8 mm). In dorsolateral view (A); dorsal (B) and ventral (C) views of specimen alive. Photographs by A. Catenazzi.

CORBIDI 18736, an adult male from near Thiuni, 13.67603 S, 70.46588 W (WGS84), 2,225 m a.s.l., Distrito Ollachea, Provincia Carabaya, Departamento Puno, Peru, collected by A. Catenazzi and A. Ttito on 14 August 2017 (Figs. 1–3).

### Paratypes

**Figure 4:**
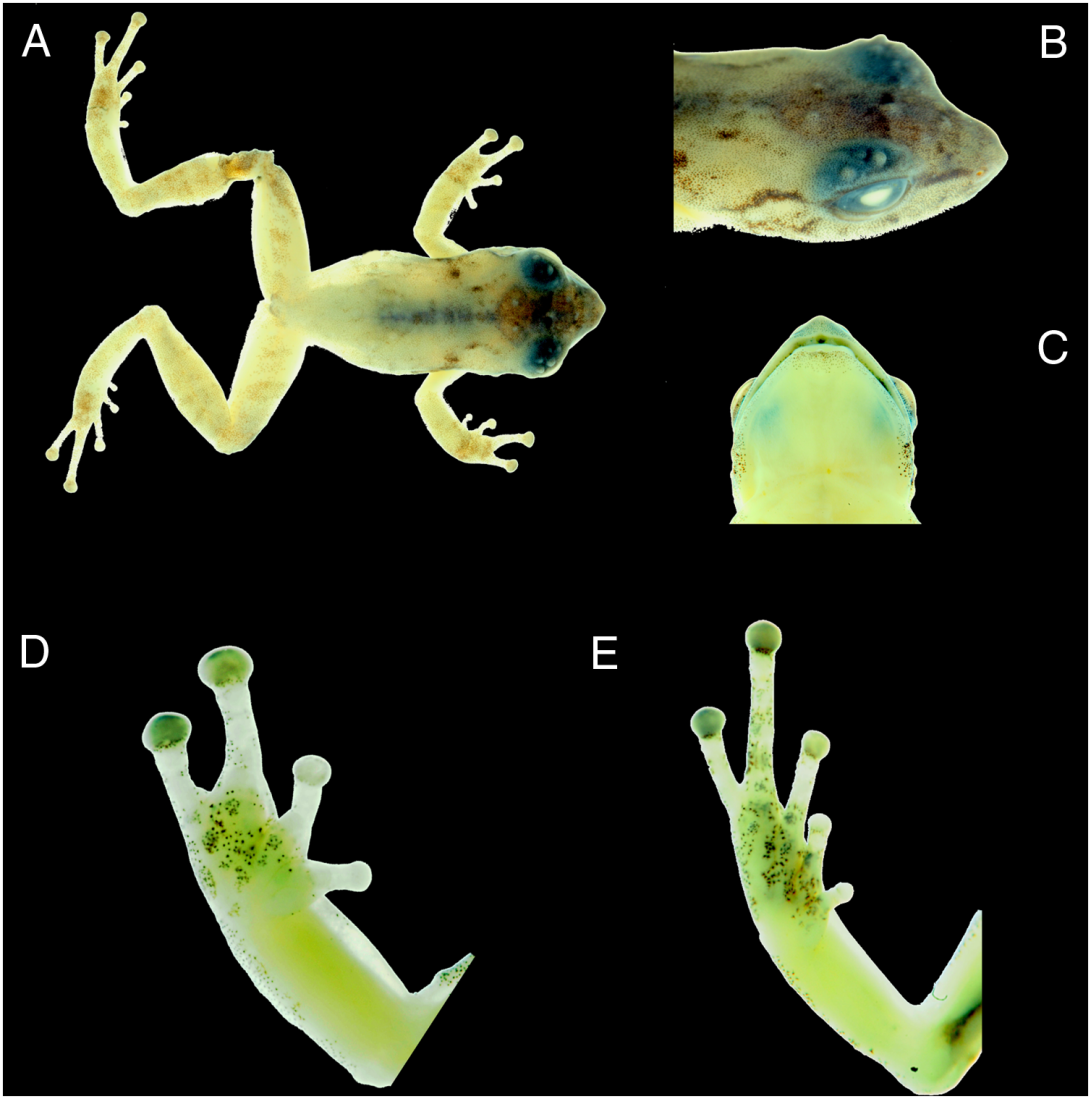
Photographs of preserved specimen of the holotype of *Pristimantis achupalla* sp. n., male CORBIDI 18736. Dorsal view of the body (A); dorsolateral view of head (B); ventral view of head (C); ventral view of hand (D) and foot (E). (hand length 3.4 mm, foot length 5.2 mm).

Three total: two adult males, CORBIDI 18730 and, MUBI 17604, and one juvenile, MUBI 17605 collected at the type locality by A. Catenazzi and A. Ttito on 14 August 2017 (Fig. 4).

### Definition

The new species is distinguished by the following combination of characters: (1) skin on dorsum rugose, that on venter areolate, discoidal fold absent, dorsolateral folds absent; (2) tympanic membrane differentiated, tympanic annulus visible, slightly robust supratympanic fold covering dorsal and posterior edges of tympanum; (3) snout acuminate in dorsal view, truncated and posteroventrally inclined in lateral view, canthus rostralis weakly concave in dorsal view, angular in lateral view, loreal region concave, rostral papilla present; (4) upper eyelid bearing two or three subconical tubercles and some rounded tubercles, cranial crests absent, and postrical turbercles present; (5) dentigerous process of vomers absent; (6) males with vocal sacs and vocal slits, nuptial excrescences absent; (7) Fingers I and II of equal length, fingers II and III bearing rounded discs about 1.2 times wider than digits, Finger IV bearing a rounded disc about twice as wide as its digit; (8) fingers with narrow lateral fringes; (9) antebrachial tubercle present; (10) ulnar and tarsal tubercles present (11) inner metatarsal tubercle oval twice as long as round outer metatarsal tubercle, low supernumerary plantar tubercles at the base of toes I, II, and III; (12) toes with narrow lateral fringes, webbing absent, Toe V longer than Toe III; (13) in life, males with dorsum brownish green; canthal stripe brown extending to the orbits; dorsolateral stripe brown; throat and belly pale green; groins, posterior surfaces of thighs, and shanks bright pale green; iris bronze with fine black reticulations; (14) SVL in adult males 10–12.8 mm; SVL in females unknown.

### Comparisons

*Pristimantis achupalla* sp. n. is morphologically similar to *P. acuminatus*, *P. amaguanae, P. bromeliaceus*, *P. enigmaticus*, *P. galdi, P. lacrimosus (variable)*, *P. limoncochensis*, *P. mendax*, *P. moro*, *P. olivaceus*, *P. omeviridis*, *P. padiali*, *P. pardalinus*, *P. pluvialis*, *P. rhodostichus*, *P. schultei*, and *P. tantanti* in having the head, body slightly compressed dorso–ventrally, and a rostral papilla or tubercle, but differs from all of them by its smaller snout-vent length of 12.8 mm, dorsal skin rugose with small, dispersed tubercles, extending to the posterior surfaces of thighs and shanks, and the presence of large tubercles on the upper eyelid.

A total of seven species resemble *P. achupalla* superficially, but can be distinguished by the following characters (in parenthesis for *P. achupalla*): *P. amaguanae* has supratympanic fold (absent); *P. bromeliaceus* has a smooth dorsum (rugose with small tubercles), and no dermal fold in the occipital/scapular absent (present sigmoidal); *P. schultei* has finely rugose dorsal skin (rugose with tubercles), and green, yellowish or orange brown dorsum (uniformly green brownish); *P. limoncochensis* lacks tympanic membrane and tympanic annulus (present), has a smooth dorsum without dorsolateral folds (rugose, present), a small tarsal fold (absent), and is lacks upper eyelid tubercles (present); *P. omeviridis* has small dentigerous processes of vomers (absent), and lacks tubercles on upper eyelid (present); *P. tantanti* has small dentigerous processes of vomers (absent), elongated ulnar tubercles (absent), dorsal skin shagreen with scattered minute tubercles (rugose with dispersed tubercles); *P. enigmaticus* has a tarsal fold (absent), dorsal skin smooth (rugose), and lacks upper eyelid tubercles (present).

#### Description of holotype

Adult male (CORBIDI 18736) with a SVL of 12.8 mm; head narrower than body, its length 40.0% of SVL; head slightly longer than wide; head width 40% of SVL; snout short, snout acuminate in dorsal view and truncated in lateral view, rostral papilla present (Fig. 3); eye large, 10% of head length, its diameter 1.3 times its distance from the nostril; nostrils slightly protuberant, situated close to snout; canthus rostralis weakly concave in dorsal view, rounded in profile; loreal area flat; lips rounded not flared; dorsal surface of head rugose and upper eyelid bearing two or three subconical tubercles and some rounded tubercles; upper eyelid width 72% of interorbital distance; cranial crest absent; supratympanic fold absent; tympanic membrane differentiated, tympanic annulus visible; tympanum diameter 23% of eye diameter; postrictal ridges or tubercles present. Choanae round, very small, positioned far anterior and laterally, widely separated from each other, not concealed by palatal shelf of maxilla; dentigerous processes of vomer and vomerine teeth barely noticeable.

Skin texture on dorsum and flanks rugose with small dispersed tubercles; no dorsolateral folds; skin on ventral surfaces and gular regions areolate; pectoral and discoidal folds absent; cloacal sheath absent, cloaca not protuberant; cloacal region lacking tubercles. Ulnar tubercle present, minute; palmar tubercle flat and bifurcate, its inner lobe much larger than outer lobe; palmar tubercle approximately twice the size of elongate, thenar tubercle; supernumerary palmar tubercles present; subarticular tubercles prominent, ovoid in ventral view rounded in lateral view; fingers with narrow lateral fringes; fingers length when adpressed, III > IV > II > I (Fig. 4); tips of digits broadly expanded and elliptical, pads with well-defined circumferential grooves (Fig. 4); forearm without tubercles. Tibia length 50.8% of SVL; foot length 40.0% of SVL; upper and posterior surfaces of hindlimbs rugose with small dispersed tubercles; heel without tubercles; outer surface of tarsus with tubercles; inner metatarsal tubercle ovoid, of higher relief and about 2.5 times the size of conical, rounded outer metatarsal tubercle; supernumerary plantar tubercles present; subarticular tubercles rounded, ovoid in dorsal view; toes without narrow lateral fringes, basal webbing absent; discs of toes expanded, rounded; toes with ventral pads well-defined by circumferential grooves; toe lengths, when adpressed, IV > V > III > II > I (Fig. 4).

Measurements of holotype (in mm): SVL 12.8, TL 6.5, FL 5.2, HL 5.1, HW 4.6, ED 1.3, TD 0.3, IOD 1.8, EW 1.3, IND 1.2. Proportions as follows: TL/SVL 0.4, FL/SVL 0.4, HL/SVL 0.4, HW/SVL 0.4, HW/HL 0,9, EW/IOD 0.69.

### Coloration of holotype in life

Dorsum brownish green (Fig. 3). Interorbital bar dark brown, forming a triangular shape posteriorly; canthus rostralis dark greenish brown; dark green on upper eyelids. Hind legs and forelimbs green barring with transverse brown blurred bars. Throat and venter pale green. The iris is bronze with dark-brown reticulations.

### Coloration of holotype in preservative

Dorsal surfaces of head brown, dorsal surface of body is cream, with slightly dark brown regions around scapulae (Fig. 4). Interorbital bar as a brown blotch that extends anteriorly and posteriorly as a mask; canthus rostralis dark brown. Upper eyelids dark bluish. Dorsal surfaces of hind limbs with dark transverse bars blurred. Iris dark gray. Throat, chest, and belly pale white to cream; ventral surfaces of thighs the color is pale green; plantar and palmar surfaces and tips of digits pale green, tubercles darker gray.

#### Variation

**Figure 5:**
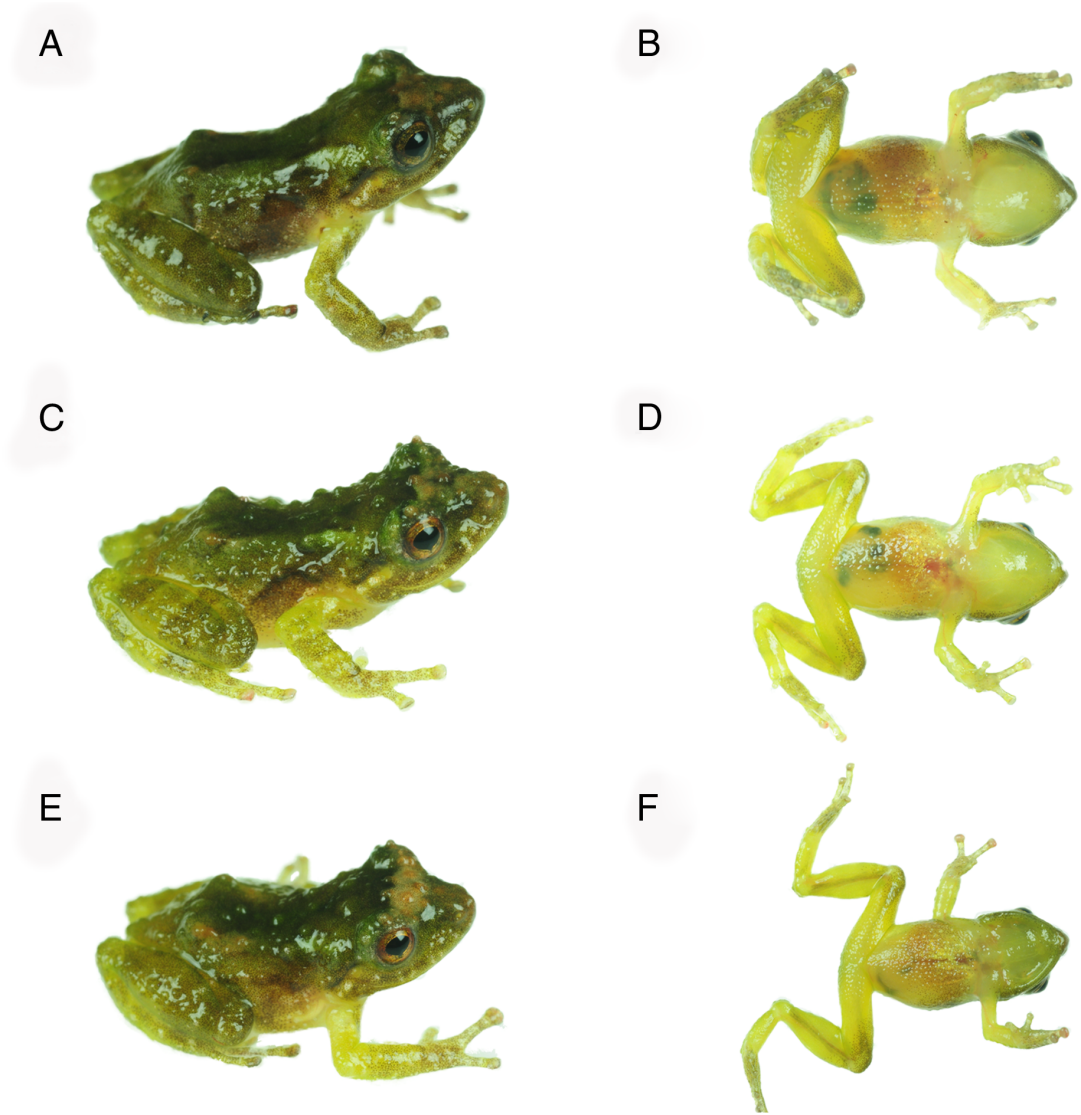
Dorsal and ventral views of *Pristimantis achupalla* sp. n. paratopotypes. Male CORBIDI 18737, SVL = 11.7 mm (A–B); male MUBI 17604, SVL = 10.0 mm (C–D); juvenile MUBI 17605, SVL = 10.1 mm (E–F). Photographs by A. Catenazzi.

**Figure 6:**
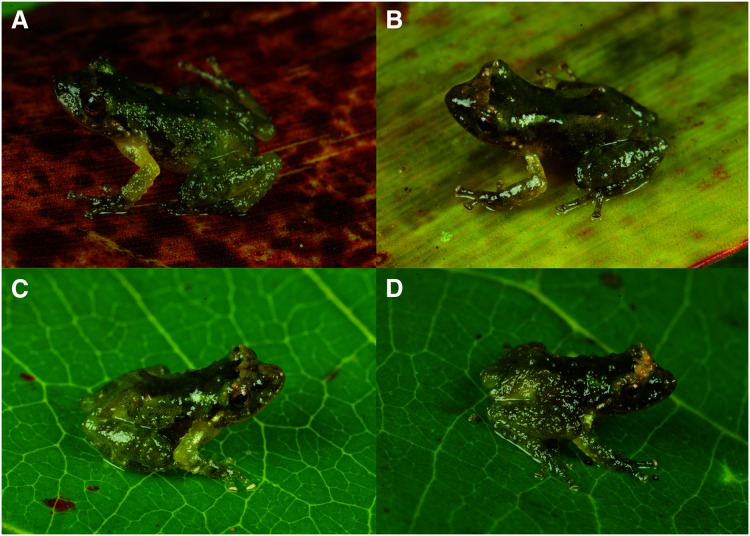
Dorsolateral views of holotype and three paratypes of *Pristimantis achupalla* sp. n. showing detail of coloration patterns and morphological feactures. Male CORBIDI 18736 (A), SVL = 12.8 mm. Male CORBIDI 18737 (B), SVL = 11.7 mm. Male MUBI 17604 (C), SVL = 10.0 mm. Juvenile MUBI 17605 (D), SVL = 10.4 mm Photographs by A. Catenazzi.

The SVL of paratypes (all males) are (in mm): CORBIDI 18737 = 11.7, MUBI 17604 = 10.0, MUBI 17605 = 10.4 (Table 1). A brown to a cream interorbital bar is present in CORBIDI 18737, MUBI 17604 and MUBI 17605 (Figs. 5–6). CORBIDI 18737 and MUBI 17604 possess faint brown barring on hind legs. Dorsal skin is generally rough whit scattered tubercles (CORBIDI 18737 just rough), indicating that skin texture might be a variable trait (Guayasamin et al., 2015).

#### Etymology

The name of the new species is a Quechua noun, used in apposition, “achupalla” = bromeliads, in reference to the use of bromeliads as its microhabitats.

#### Distribution, natural history and threats

We found four specimens of *Pristimantis achupalla* by inspecting epiphytic bromeliads in a relictual cloud forest along the Macusani-San Gabán road (Ollachea Valley), which connects the Peruvian Altiplano to the Amazon rainforest and the interoceanic highway between Peru and Brazil. The relictual montane forest covers a small ridge and is accessible through a maintenance trail for a high-voltage power line (Catenazzi & Ttito, 2018, 2019). We found the holotype and male CORBIDI 18737 in the same bromeliad, a medium-sized bromeliad on a tree branch overhanging the power maintenance trail, along with a third specimen that escaped capture during the inspection. Following the discovery of these individuals at 16 h 20, we haphazardly searched several bromeliads along the trail and around the high-voltage power pole until 17 h 00, and found the two additional specimens (MUBI 17604 and MUBI 17605), as well as a juvenile of *Gastrotheca* cf. *testudinea*. On the basis of microhabitats associated with all four specimens, and the flattened body shape of head and body allowing frogs to slide between leaves, we hypothesize that *P. achupalla* are bromeliad specialists. Searching bromeliads, we surveyed the leaf litter and found *Pristimantis platydactylus*, and the two recently described species *Psychrophrynella glauca* and *Noblella thiuni* (Catenazzi & Ttito, 2018, 2019). We suggest classifying *P. achupalla* sp. n. as “Data Deficient” according to the IUCN red list categories and criteria (IUCN Standards & Petitions Committee, 2019) based on the limited information on its geographic range and population abundance, and due to the known threats of deforestation, small-scale agriculture, as well as hydropower, road construction, and similar large-scale projects affecting the upper Ollachea Valley.

## Discussion

The previously defined *Pristimantis lacrimosus* species group was non-monophyletic group (Rivera-Correa & Daza, 2016, 2020; Gonzalez-Duran et al., 2017; Ron et al., 2020), but its redefinition by Ron et al. (2020), who included *Pristimantis lacrimosus* in their matrix, corresponds with clade B, *sensu* Rivera-Correa & Daza (2016); and includes *P. acuminatus*, *P. amaguanae, P. aureolineatus, P. bromeliaceus*, *P. crucifer*, *P. ecuadorensis*, *P. enigmaticus*, *P. eremitus*, *P. galdi, P. jorgevelosai, P. lacrimosus*, *P. limoncochensis*, *P. mendax*, *P. mindo, P. nankints, P. nyctophylax*, *P. olivaceus*, *P. omeviridis*, *P. ornatissimus*, *P. pluvialis*, *P. pulchridormientes*, *P. romeroae*, *P. schultei, P. subsigillatus, P. tantanti, P. zorro* (Ron et al., 2020; Rivera-Correa & Daza, 2020*)*. Currently, the distribution of *P. lacrimosus* species group comprehends Central America, Colombia, the Guianas, Ecuador, Peru and Bolivia. We associated *a priori P. achupalla* with the *P. lacrimosus* group for sharing some synapomorphies proposed by Padial et al., (2104), such as a dorsoventrally compressed head and body, upper eyelids bearing large tubercles, and presence of a rostral papilla. Our phylogenetic analysis suggests with moderate to strong support that *P. achupalla* is more closely related to one undescribed *Pristimantis* species from the montane forest of Cusco, and both species are related to species of the *P. lacrimosus* group *sensu* Ron et al. (2020). Furthermore, we obtained a topology that is relatively consistent with previous studies (Rivera-Correa & Daza, 2016, 2020; Gonzalez-Duran et al., 2017; Ron et al., 2020). However, some nodes present low support, probably because it is based on only one genetic marker, in addition to the known challenges with alignment of 16S rRNA. Furthermore, existing gaps in the distribution of *P. lacrimosus* group continue to affect the reconstruction of the evolutionary history of this group (Rivera-Correa & Daza, 2020*)*.

On the other hand, the analysis of genetic distances (uncorrected *p*-distances) shows that *Pristimantis achupalla* has moderate genetic distance (*p*-distance, 0.05–0.06 respectively, Table 2) with *Pristimantis* sp. from Cusco, and *P. amaguanae* from Pastaza, Ecuador (Ron et al., 2020). Although there is no fixed threshold for delimiting species, genetic distances among closely related strabomantid species often exceed 0.03, and for 16S, a distance of 0.03 is a reasonable criterion to identify putative new species (Fouquet et al., 2007; Chávez, García-Ayachi & Catenazzi, 2021). *Pristimantis achupalla*, *Pristimantis* sp. and *P. amaguanae* also show low morphological differences and share similar mircrohabitat and presumably ecological niche, but different geographical space. Other closely related species are *P. moro* and *P. bromeliaceus*, which are larger than *P. achupalla*. In contrast, other species such as *P*. cf. *olivaceus, P. pluvialis* and *P. pulchridormientes*, show higher genetic distances between 10% and 17%. *Pristimantis achupalla* occurs in remnant of montane forest at 2225 m a.s.l. in the Ollachea valley, part of the Cordillera de Carabaya. Recent herpetological surveys in the Cordillera de Carabaya have discovered several new species and allocated species to new genera (see De la Riva et al., 2017; Catenazzi & Ttito, 2018; Catenazzi & Ttito, 2019). Moreover, *P. achupalla* together with *P. olivaceus* (Peru and Bolivia) represent the most southern distribution of the *P. lacrimosus* group. Most suitable areas for terraranas in the Andes of Peru and Bolivia have yet to be explored, and there are good reasons to suspect that many new species will be found (Lehr & Catenazzi, 2009; De la Riva et al., 2017; Catenazzi & Ttito, 2019). Futhermore, the Cordillera de Carabaya appears to host substantial beta diversity of Holadeninae and Strabomantinae, suggesting an intriguing and underappreciated evolutionary history for this groups in southern Peru.

## Conclusions

We describe a new species of terrestrial breeding frog in the genus *Pristimantis*. We justify the generic location-based on morphological similarity and phylogenetic analyses. The new species *P. achupalla* is nested within the *P. lacrimosus* group and is closely related to an undescribed species *P*. sp. from Peru, *P. amagunae, P*. sp. from Ecuador, and *P. bromeliaceus*. These species of minute, bromeliad-living frogs form a clade well supported in the phylogeny. Additionally, several meristic traits distinguish *P. achupalla* from similar species in *P. lacrimosus* group. We discuss the difficulty of justifying the position of *P. achupalla* within groups such as *P. lacrimosus*, but we also confirm the evolutionary unicity of *P. achupalla* which supports our taxonomic decision to describe it as new species. Our contribution increases the knowledge of the rich diversity of terrestrial breeding frogs found at high elevations on the eastern slopes of the Cordillera de Carabaya.

## Supplemental Information

10.7717/peerj.11878/supp-1 Supplemental Information 1 Specimens examined. List of specimens of species examined for this work, collections abbreviation: CORBIDI = Herpetology Collection, Centro de Ornitología y Biodiversidad, Lima, Peru; AMNH = American Museum of Natural History, New York, USA; USNM = Smithsonian Institution, National Museum of Natural History, Washington, USA; MUSM = Museo de Historia Natural, Universidad Nacional Mayor de San Marcos, Lima, Peru; MHNC = Museo de Historia Natural, Universidad San Antonio Abad del Cusco, Cusco, Peru.

10.7717/peerj.11878/supp-2 Supplemental Information 2 Gene sequences for molecular analyses. GenBank accession numbers for the taxa and genes sampled in this study. Voucher ROM 43978 was previously identified as *Pristimantis zeuctotylus* by Hedges et al. (2008a), but is treated herein as *Pristi­mantis* sp. following Padial et al. (2014).

10.7717/peerj.11878/supp-3 Supplemental Information 3 *Pristimantis achupalla* sp. n. 16S rRNA sequences.

## Additional Information and Declarations

### Field Study Permissions

The following information was supplied relating to field study approvals (*i.e*., approving body and any reference numbers): Collecting permits were issued by the Dirección General Forestal y de Fauna Silvestre, Ministerio de Agricultura y Riego (permits #0292-2014-MINAGRI-DGFFS/DGEFFS, #029-2016-SERFOR-DGSPFS).

### DNA Deposition

The following information was supplied regarding the deposition of DNA sequences: The fragment of the 16S rRNA mitochondrial gene sequences are available in the Supplemental Files and at GenBank: MW724812, MW724813, MW724814, MW724815 and MW817091.

### Data Availability

The following information was supplied regarding data availability: Holotype (CORBIDI 18736) is deposited at CORBIDI - Herpetology Collection, Centro de Ornitología y Biodiversidad, Lima, Peru. The gene sequences for molecular analyses are available in the Supplemental File.

### New Species Registration

The following information was supplied regarding the registration of a newly described species: Publication LSID: urn:lsid:zoobank.org:pub:88FB4422-449E-45BF-B2E3-0A30D00090B8. *Pristimantis achupalla* sp. n. LSID: urn:lsid:zoobank.org:act:D9EFDD3D-F0C3-40D8-BEBF-71372B43D42D.
