# Supplementary material for: Pristimantis achupalla sp. n., a new minute species of direct-developing frog (Amphibia, Anura, Strabomantidae) inhabiting bromeliads of the montane forest of the Amazonian Andes of Puno, Peru
Source: PeerJ. 2021 Sep 23;9:e11878. doi: 10.7717/peerj.11878 (PMC8466079; doi:10.7717/peerj.11878)
Supplement: Supplemental Information 1 — List of specimens of species examined for this work, collections abbreviation: CORBIDI = Herpetology Collection, Centro de Ornitología y Biodiversidad, Lima, Peru; AMNH = American Museum of Natural History, New York, USA; USNM = Smithsonian Institution, National Museum of Natural History, Washington, USA; MUSM = Museo de Historia Natural, Universidad Nacional Mayor de San Marcos, Lima, Peru; MHNC = Museo de Historia Natural, Universidad San Antonio Abad del Cusco, Cusco, Peru. [file peerj-09-11878-s001.docx]

**Appendix I.**

List of specimens of species examined for this work, collections abbreviation: CORBIDI = Herpetology Collection, Centro de Ornitología y Biodiversidad, Lima, Peru; AMNH = American Museum of Natural History, New York, USA; USNM = Smithsonian Institution, National Museum of Natural History, Washington, USA; MUSM = Museo de Historia Natural, Universidad Nacional Mayor de San Marcos, Lima, Peru; MHNC = Museo de Historia Natural, Universidad San Antonio Abad del Cusco, Cusco, Peru.

*Pristimantis acuminatus* (2 specimens): PERU: Amazonas: Quebrada Kampankis,

CORBIDI 11388, 11403.

*Pristimantis bromeliaceus* (8 specimens): PERU: Amazonas: Chonza Alta, Bagua,

CORBIDI 778; Pasco: Comunidad Campesina Chacos, CORBIDI 3859; San

Martín: Abra Patricia, CORBIDI 510–12, 516–17; Quintecocha, MUSM

24448–49.

*Pristimantis* cf. *tantanti* (3 specimens): PERU: Cusco: Cashiriari-3, S of Río Camisea, USNM 537763; Pagoreni, Río Camisea, USNM 537764; San Martín-3, ~5 km N Río Camisea, USNM 537762.

*Pristimantis lacrimosus* (10 specimens): PERU: Loreto: Sierra del Divisor, CORBIDI

3941; Río Tapiche, CORBIDI 12133–38; Campamento Piedras, Putumayo,

CORBIDI 5894, 5899, 5903.

*Pristimantis olivaceus* (14 specimens): PERU: Cusco: Cashiriari-2, ~4 km S of Río

Camisea, USNM 538039–43; Cashiriari-3, S of Río Camisea, USNM 537805;

Konkariari Creek Camp, Río Urubamba, USNM 538044–45; Comunidad Nativa

Puyentimari, CORBIDI 8296, 9765–66, Kinteroni, CORBIDI 10260; Madre

de Dios: Colpa de Guacamayos, Río Tambopata, USNM 332440; Pakitza, PN

Manu, USNM 342614–15.

*Pristimantis pluvialis* (6 specimens): PERU: Cusco: Paucartambo: Cusco-Pilcopata

road, 1480 m, AMNH 157016; USNM 345921, 346336; MHNC 15489–90.

*Pristimantis rhodostichus* (2 specimens): PERU: Amazonas: Cabeceras Katerpiza, CORBIDI 9441; Loreto: Cabeceras Wee, CORBIDI 11430.

*Pristimantis schultei* (21 specimens): PERU: Amazonas: Laguna de los Cóndores, MUSM 23040–48; ACP Huiquilla, CORBIDI 368; Yuramarca, CORBIDI 452–62.
