# Supplementary material for: Pristimantis achupalla sp. n., a new minute species of direct-developing frog (Amphibia, Anura, Strabomantidae) inhabiting bromeliads of the montane forest of the Amazonian Andes of Puno, Peru
Source: PeerJ. 2021 Sep 23;9:e11878. doi: 10.7717/peerj.11878 (PMC8466079; doi:10.7717/peerj.11878)
Supplement: Supplemental Information 2 — GenBank accession numbers for the taxa and genes sampled in this study. Voucher ROM 43978 was previously identified as Pristimantis zeuctotylus by Hedges et al. (2008a), but is treated herein as Pristi­mantis sp. following Padial et al. (2014). [file peerj-09-11878-s002.docx]

**Appendix II.**

**Gene sequences for molecular analyses**

GenBank accession numbers for the taxa and genes sampled in this study. Voucher ROM 43978 was previously identified as *Pristimantis zeuctotylus* by Hedges et al. (2008a), but is treated herein as *Pristi­mantis* sp. following Padial et al. (2014).

| ***Species*** | **Voucher** | **16S** |
| --- | --- | --- |
| *Niceforonia dolops* | -- | EF493394.1 |
| *P. acerus* | KU 217786 | EF493678.1 |
| *P. achupalla sp. n.* | MUBI 17605 | MW724812 |
| *P. achupalla sp. n.* | MUBI 17604 | MW724813 |
| *P. altamazonicus* | KU 215460 | EF493670.1 |
| *P. angustilineatus* | UVC 15828 | JN371034.1 |
| *P. appendiculatus* | KU177637 | EF493524.1 |
| *P. aureolineatus* | QCAZ 42286 | MT636530 |
| *P. boulengeri* | MHUAA 8951 | KU724435.1 |
| *P. brevifrons* | nrps 0059 | JN991433.1 |
| *P. bromeliaceus* | QCAZ 16699 | MT636527 |
|  | QCAZ 62940 | MT636523 |
| *P. calcarulatus* | KU 177658 | EF493523.1 |
| *P. cedros* | MZUTI 1713 | KT210155.1 |
| *P. cf. mendax* | MTD 45080 | EU186659.1 |
| *P. cf. olivaceus* | MNP-P6V74067 | MW075410.1 |
|  | CORBIDI 17473 | KX155579 |
|  | MUBI 17676 | MW724814 |
| *P. cosnipatae* | MHNG 2606.72 | MW724815 |
| *P. crucifer* | KU 177733 | EU186718.1 |
| *P. danae* | MVZ 272358 | KY652661 |
| *P. diadematus* | KU 221999 | EU186668.1 |
| *P. dorsopictus* | MHUAA7638 | KP082874.1 |
| *P. ecuadorensis* | CJ 5350 | KX785343 |
|  | CJ 5351 | KX785344 |
| *P. enigmaticus* | QCAZ 40918 | MT636520 |
| *P. erythropleura* | nrps 0055 | JN991445.1 |
| *P. galdi* | QCAZ 32368 | EU186670.1 |
| *P. glandulosus* | KU 218002 | EF493676.1 |
| *P. imitatrix* | KU 215476 | EF493667.1 |
| *P. jaguensis* | MHUAA 7249 | KP082870.1 |
| *P. jorgevelosai* | JDL 26123 | DQ195461.1 |
| *P. lacrimosus* | QCAZ 55238 | MT636518 |
|  | QCAZ 59474 | MT636517 |
|  | QCAZ 40261 | MT636524 |
|  | QCAZ 59469 | MT636516 |
| *P. lindae* | MUSM 27902 | KY652653 |
| *P. limoncochensis* | QCAZ 43794 | MT636525 |
|  | QCAZ 19180 | MT636532 |
| *P. melanogaster* | -- | EF493664.1 |
| *P. mindo* | MZUTI 1382 | KF801584.1 |
|  | MZUTI 1381 | KF801583.1 |
|  | QCAZ 56512 | MT636522 |
|  | MZUTI 1756 | KF801581.1 |
|  | QCAZ 42197 | MT636531 |
| *P. moro* | AJC 1860 | JN991454.1 |
|  | AJC 1753 | JN991453.1 |
| *P. nankints* | QCAZ 69137 | MT636514 |
| *P. nyctophylax* | KU 177812 | EF493526.1 |
|  | QCAZ 32288 | MT636519 |
| *P. omeviridis* | QCAZ 10564 | MK881398 |
|  | QCAZ 19664 | EU13057 |
| *P. ockendeni* | RVM 5.12 | KY652654 |
| *P. orcesi* | KU 218021 | EF493679.1 |
| *P. ornatissimus* | MZUTI 4798 | KU720463 |
|  | MZUTI 4806 | KX785341 |
|  | MZUTI 4807 | KX785342 |
| *P. pahuma* | MZUTI 493 | KT210158.1 |
| *P. petrobardus* | KU212293 | EF493367.1 |
| *P. pharangobates* | MVZ 272360 | KY652655 |
| *P. platydactylus* | MNCN 5524 | EU192255.1 |
| *P. pluvialis* | CORBIDI 11862 | KX155577 |
|  | CORBIDI 16695 | KX155578 |
| *P. pulchridormientes* | CORBIDI 15566 | KX664107 |
|  | CORBIDI 15563 | KX664106 |
| *P. pulvinatus* | KU 181015 | EF186723.1 |
| *P. pycnodermis* | KU 218028 | EF493680.1 |
| *P. reichlei* | CORBIDI 16219 | KY652657 |
| *P. ridens* | AMNHA 124551 | EF493355.1 |
| *P. romeroae* | QCAZ 41121 | MT636528 |
| *P. rubicundus* | QCAZ 58932 | MT372670 |
| *P. salaputium* | MUSM 27916 | KY652658.1 |
| *P. schultei* | KU 212220 | EF493681.1 |
| *Pristimantis* sp. | CORBIDI 12183 | MW817091 |
| *P. subsigillatus* | MECN 10117 | KF801580.1 |
| *P. toftae* | AC 107.07 | KY652659 |
| *P. urani* | MHUAA 7471 | KU724442.1 |
| *P. viejas* | EMM247 | JN991477.1 |
| *Pristimantis sp.* | ROM 43978 | EU186678.1 |
|  | KU 291702 | EF493351.1 |
|  | QCAZ 60398 | MT636515 |
|  | QCAZ 58956 | MT636526 |
| *P. zophus* | nrps 0072 | JN991478.1 |
| *P. zorro* | MHUAA 8816 | MT747835 |
|  | MHUAA 8814 | MT747834 |
